# Dissection of closely linked QTLs controlling stigma exsertion rate in rice by substitution mapping

**DOI:** 10.1007/s00122-021-03771-9

**Published:** 2021-01-25

**Authors:** Quanya Tan, Chengshu Wang, Xin Luan, Lingjie Zheng, Yuerong Ni, Weifeng Yang, Zifeng Yang, Haitao Zhu, Ruizhen Zeng, Guifu Liu, Shaokui Wang, Guiquan Zhang

**Affiliations:** Guangdong Provincial Key Laboratory of Plant Molecular Breeding, State Key Laboratory for Conservation and Utilization of Subtropical Agro-Bioresources, South China Agricultural University, Guangzhou, 510642 China

## Abstract

**Key message:**

Through substitution mapping strategy, two pairs of closely linked QTLs controlling stigma exsertion rate were dissected from chromosomes 2 and 3 and the four QTLs were fine mapped.

**Abstract:**

Stigma exsertion rate (SER) is an important trait affecting the outcrossing ability of male sterility lines in hybrid rice. This complex trait was controlled by multiple QTLs and affected by environment condition. Here, we dissected, respectively, two pairs of tightly linked QTLs for SER on chromosomes 2 and 3 by substitution mapping. On chromosome 2, two linkage QTLs, *qSER-2a* and *qSER-2b*, were located in the region of 1288.0 kb, and were, respectively, delimited to the intervals of 234.9 kb and 214.3 kb. On chromosome 3, two QTLs, *qSER-3a* and *qSER-3b*, were detected in the region of 3575.5 kb and were narrowed down to 319.1 kb and 637.3 kb, respectively. The additive effects of four QTLs ranged from 7.9 to 9.0%. The epistatic effect produced by the interaction of *qSER-2a* and *qSER-2b* was much greater than that of *qSER-3a* and *qSER-3b*. The open reading frames were identified within the maximum intervals of *qSER-2a*, *qSER-2b* and *qSER-3a*, respectively. These results revealed that there are potential QTL clusters for SER in the two regions of chromosome 2 and chromosome 3. Fine mapping of the QTLs laid a foundation for cloning of the genes of SER.

**Supplementary Information:**

The online version contains supplementary material available at 10.1007/s00122-021-03771-9.

## Introduction

Rice is one of the most important staple food crops in the world. In the past decades, great progress has been made in improving rice yield by the utilization of heterosis in hybrid rice (Yuan 2017). The male sterility line (MSL) is a key line of the hybrid rice system. Since the cultivated rice is mainly self-pollination (Virmani and Athwal 1973), improving the outcrossing ability of MSLs is very important for hybrid rice seed production. Stigma exsertion can improve the outcrossing ability of MSLs by catching more pollens from male parents (Marathi and Jena 2015). Therefore, stigma exsertion rate (SER) is an important trait of outcrossing in hybrid rice. MSLs with high SER can produce more hybrid seeds in hybrid rice seed production (Virmani 1994).

In the past two decades, dozens of QTLs responsible for the SER have been identified from rice germplasm resources (Marathi and Jena 2015; Liu et al. 2019; Tan et al. 2020). Because of high SER, MSLs or their maintainer lines were usually used to detect QTLs for SER. From the cytoplasmic male sterile (CMS) maintainer line XieqingzaoB, *qSE7* was located on chromosome 7 (Zhang et al. 2018), and *qSE11* was mapped on chromosome 11 (Rahman et al. 2017). From the CMS maintainer line II-32B, *qSER-7* was identified on chromosome 7 (Liu et al. 2019). The wild rice is the important germplasm resources with strong outcrossing ability (Marathi et al. 2015; Marathi and Jena 2015). Many QTLs for SER were identified from *O. rufipogon* (Xiong et al. 1999; Li et al. 2001; Uga et al. 2003a; Huang et al. 2012; Bakti and Tanaka 2019; Zou et al. 2020), *O. longistaminata* (Li et al. 2010), *O. glumaepatula* (Tan et al. 2020), *O. barthii* and *O. meridionalis* (Zou et al. 2020). Many of the QTLs for SER identified from different mapping populations and different mapping methods were found to be position overlapping in rice genome (Tan et al. 2020). The most common and most powerful QTLs for SER were frequently detected on the short arm of chromosome 3. In the region of about 12.0–17.0 Mb of chromosome 3, seven QTLs for SER, *qES3* (Yamamtot et al. 2003; Miyata et al. 2007), *PES-3* (Yue et al. 2009), *qSPE3* (Feng et al. 2010), *qSSE3* (Li et al. 2014), *qSERb3-1* and *qSERm3-1* (Zou et al. 2020), and *qSER-3a* (Tan et al. 2020) were detected. *GS3*, a gene controlling seed length (Fan et al. 2006) with pleiotropic effects on stigma length and stigma exsertion (Takano-Kai et al. 2011; Zhou et al. 2017a), were also located in the region. In the region of about 3.0–5.4 Mb of chromosome 2, five QTLs for SER, *qPES-2* (Li et al. 2003), *qPES-2* (Deng et al. 2010), *qPES2.2* (Li et al. 2017), and *qSERr2-1* and *qSERm2-1* (Zou et al. 2020) were located. Since only one QTL was usually detected in an overlapping region in each study, it is unclear how many QTLs for SER exist in these regions. In addition, although a large number of QTLs for SER have been reported in the rice genome, few QTLs were located within the 500 kb interval and none of them has been cloned (Marathi and Jena 2015; Liu et al. 2019; Tan et al. 2020).

In recent two decades, substitution mapping has become a powerful tool for QTL detection of complex traits instead of traditional genetic mapping. Compared with primary mapping populations, such as F_2_, recombinant inbred lines (RILs), doubled haploid (DH) lines, the secondary mapping populations, such as chromosomal segment substitution lines (CSSLs), single-segment substitution lines (SSSLs), and near-isogenic lines (NILs), segregate only in target genes (segments) in the same genetic background (Eshed and Zamir 1995; Wang et al. 2012; Tan et al. 2020). For substitution mapping of QTLs in rice genome, we constructed an SSSL library (Zhang et al. 2004; Xi et al. 2006; He et al. 2017; Zhao et al. 2019; Zhang 2019). The SSSL library was widely used to detect QTLs in rice genome (Wang et al. 2012; Zhang et al. 2012; Zhao et al. 2016; Zhou et al. 2017b; Sui et al. 2019). Recently, we detected seven QTLs for SER on five chromosomes using a set of SSSLs derived from *O. glumaepatula* (Tan et al. 2020). In the present study, we used a set of SSSLs carrying substitution segments of IR66897B (IB), a CMS maintainer line, to fine map QTLs for SER. By substitution mapping, two QTLs for SER were detected in 1288.0 kb region of chromosome 2, and other two QTLs for SER were located in 3575.5 kb region of chromosome 3. The four QTLs were limited to 214.3–637.3 kb. These results revealed that these two chromosomal regions were potential QTL clusters for SER. The fine mapping of the four QTLs for SER laid the foundation for gene cloning.

## Materials and methods

### The SSSLs used

We have constructed a SSSL library with 2360 SSSLs by using 43 accessions of 7 species with rice AA-genome as donors and Huajingxian74 (HJX74), an elite *indica* variety, as recipient. Each SSSL carries only one chromosomal segment from a donor in the HJX74 genetic background (Zhang et al. 2004; Xi et al. 2006; He et al. 2017; Zhao et al. 2019; Zhang 2019). A set of 41 SSSLs carrying substitution segments from the donor of IR66897B (IB), a CMS maintainer line from International Rice Research Institute (IRRI), was selected from the HJX74-SSSL library. After surveying of SER, 7 SSSLs with higher SER than HJX74 were selected for this study (Table S1).

### Field experiments

All plant materials were planted in the farm of South China Agriculture University, Guangzhou, China (23° 07′ N, 113° 15′ E). The materials were planted in nine cropping seasons from 2014 to 2018, two cropping seasons per year. The first cropping season (FCS) was from late February to middle July, the second cropping season (SCS) was from late July to middle November. Field cultivation and controlling of diseases and insect pests followed by conventional methods in South China.

### Molecular markers and PCR protocol

SSR markers labeled “RM” were selected from online resources (https://archive.gramene.org/markers/). The “PSM” and “InDel” markers were designed using the software of Primer Premier 5.0 (Lalitha 2000) (Table S2). The *GS3* gene was genotyped by a functional marker, SF28-*Pst*I (Zhou et al. 2017a). Genomic DNA was extracted from freshly frozen leaves of plants using a reported method (Murray and Thompson 1980). Target DNA segments were amplified with the following program, 94 °C for 5 min, followed by 35 cycles of 94 °C for 30 s, 55 °C for 30 s, and 72 °C for 45 s, and a final extension of 72 °C for 5 min. The PCR products were separated on 6% denatured PAGE, and bands detected using the silver staining methods described by Fang et al. (2019).

### Phenotyping of traits and statistical analysis

Stigma exsertion (SE) was classified into single stigma exsertion (SSE) and dual stigma exsertion (DSE). SER refers to the total stigma exsertion rate, including single stigma exsertion rate and dual stigma exsertion rate. For investigating SER, the 8–10 main panicles were sampled from plants of flourishing florescence. The SER per panicle was calculated based on the visual aspect of exserted stigmas (Liu et al. 2019). Grain traits were measured by the yield traits scorer (YTS), a rice phenotypic facility (Yang et al. 2014). The arcsine square root transformation of SER value was used for statistical analysis, one-way ANOVA. Dunnett *t* test treated one group as a control, and compared all other groups against it. Least significance range (LSR) was used for multiple rang test among multiple groups (Duncan 1955). The data analysis and figure making were done by SPSS statistics 23.0 and OriginPro 9.0 (https://www.originlab.com).

### Substitution mapping of QTLs

The minimum length (*L*_min_) of a substitution segment refers to the length between two markers of donor genotype at the end of the substitution segment. The maximum length (*L*_max_) refers to the length between two markers flanking the two end of substitution segment with background genotype. The estimated length (*L*_est_) = (*L*_min_ + *L*_max_)/2 (Tan et al. 2020). Secondary SSSLs were developed by backcrossing primary SSSLs with HJX74. The QTLs for SER were mapped by the substitution mapping method (Eshed and Zamir 1995; Tan et al. 2020). When the phenotype of SER had significant difference between a SSSL and the recipient, a QTL for SER was located in the region of substitution segment of SSSL. Additive effect of a single gene was defined as half of the phenotypic difference between SSSL and HJX74. Epistatic effects among QTLs were estimated by the formula, $i=\left({P}_{n}-{P}_{0}\right)-2\sum_{i=1}^{n}\left({a}_{i}\right)$, where *i* is an epistasis among the pyramided QTLs, *P*_*n*_ is a phenotype of a pyramiding line harboring n of QTLs, *P*_0_ is a phenotype of HJX74, *a*_*i*_ (1 ≤ *i* ≤ *n*) is an additive effect of a single gene at the ith QTL. Epistatic effects among QTLs were tested in Student's *t* test under null hypothesis (H_0_) *i* = 0. QTLs were named by the method of McCouch et al. (1997). Linkage maps of markers were drawn by using MapChart2.2 (https://www.wur.nl/en/show/Mapchart.htm).

### Gene annotation in the regions of QTLs

Two rice gene annotation databases, the Rice Annotation Project Database (RAP-DB) and the Rice Genome Annotation Project of Michigan State University (MSU-RAP), were used to identify the open reading frames (ORFs) within the target QTL regions of Nipponbare reference genome (IRGSP-1.0) from the Gramene database (http://www.gramene.org/).

## Results

### SER in the SSSLs derived from the CMS maintainer line

**Fig. 1:**
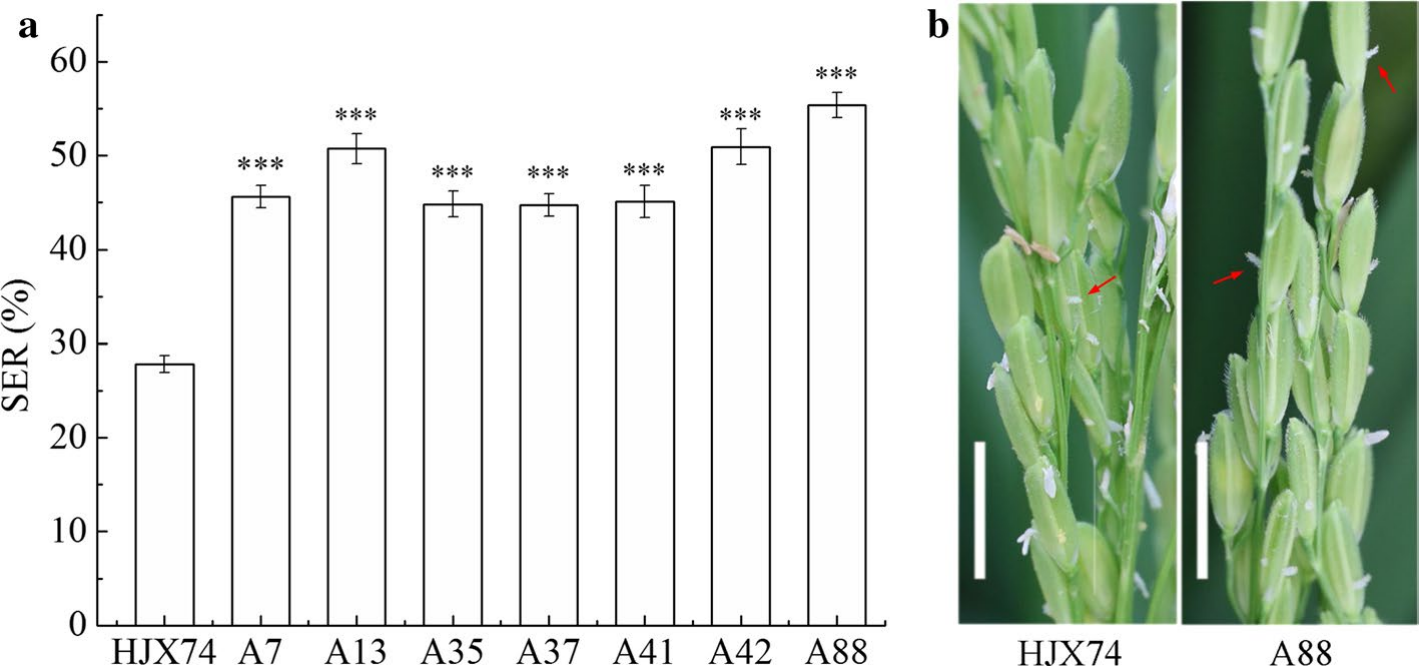
SER of seven SSSLs and HJX74. **a** SER of the SSSLs and control HJX74. Data were presented as mean ± S.E. in nine cropping seasons. Dunnett *t* test, *** *P* ≤ 0.001. **b** Exserted stigmas in the panicles of HJX74 and A88. Red arrows point the exserted stigmas. Scale bar, 1 cm. *SSSL* single-segment substitution line. *SER* stigma exsertion rate

After investigating of SER from a set of SSSLs derived from the CMS maintainer line IB, seven SSSLs with higher SER than the HJX74 recipient were selected. Then, the SER of seven SSSLs was tested in nine cropping seasons from 2014 FCS to 2018 FCS. Compared with HJX74, the seven SSSLs were showed higher SER at the *P* ≤ 0.001 level in every cropping season. Average SER of seven SSSLs in the nine cropping seasons was from 44.8 to 55.3% with 17.0–27.5% higher than that of the control HJX74 (Fig. 1 and Table S1).

The substitution segments of the seven SSSLs were detected by increasing the density of molecular markers (Table S2). The substitution segments were detected on chromosomes 2 and 3 with the estimated lengths from 1843.4 to 5162.6 kb, respectively (Table S3).

Eight agronomic traits in the seven SSSLs were investigated in two cropping seasons of 2018. Compared with HJX74, the SSSLs had no significant difference at *P* = 0.001 level in all traits, but had significant differences at *P* ≤ 0.01 level in some traits (Table S4). The results showed that the genetic background of the SSSLs was similar to HJX74 except for SER.

### Two closely linked QTLs for SER were dissected on the substitution segment of chromosome 2

**Fig. 2:**
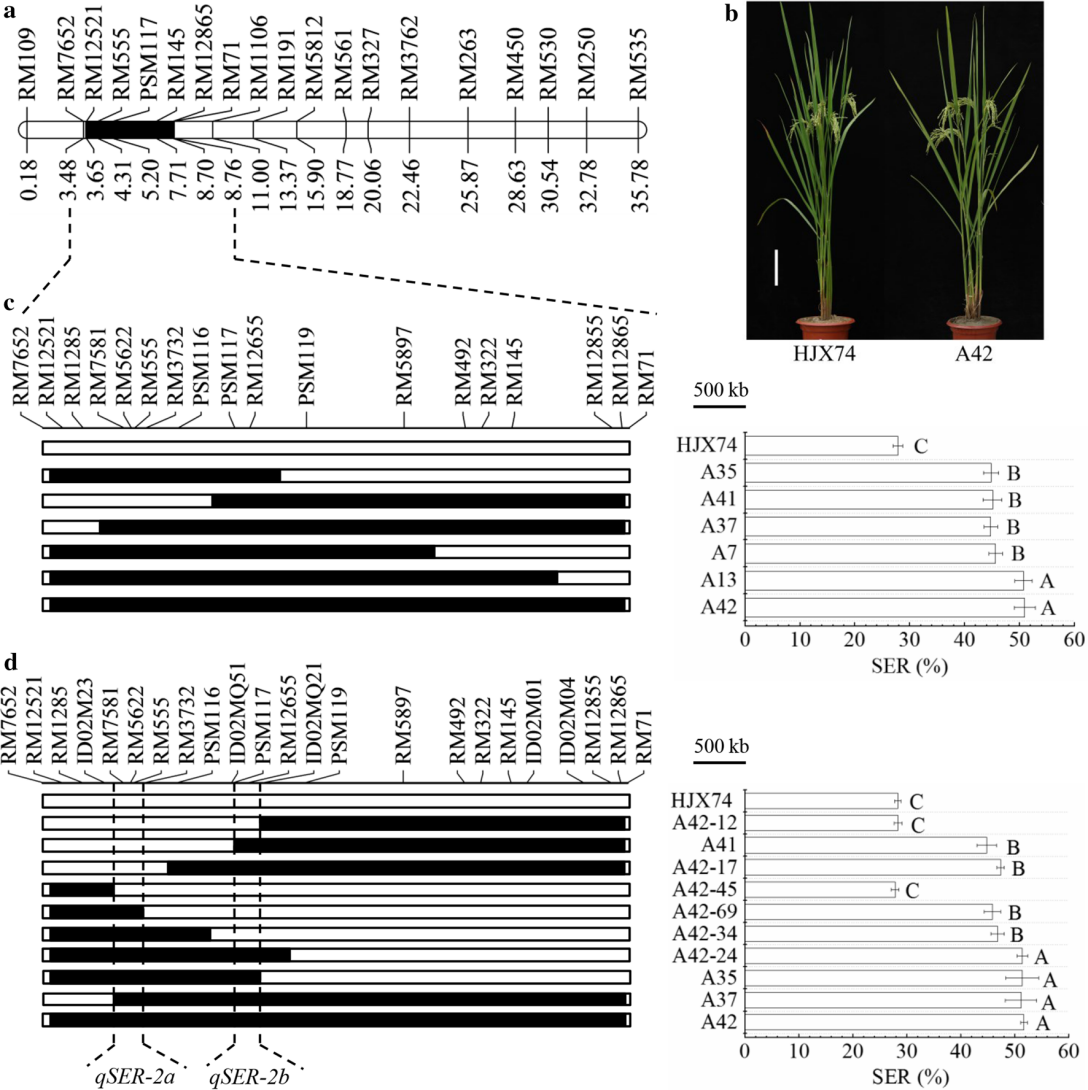
Substitution mapping of QTLs for SER on chromosome 2. **a** The substitution segment of SSSL-A42 on chromosome 2. Physical distance (Mb) was shown under the chromosome. **b** Plant type of SSSL-A42 and HJX74, scale bar, 15 cm. **c** Substitution mapping based on the substitution segments of six SSSLs. **d** Secondary substitution mapping of QTLs based on the substitution segment of SSSL-A42. *SER* stigma exsertion rate. SER (%) was a mean ± S.E. in nine cropping seasons (**c**) and two cropping seasons (**d**). Different alphabet letters denote differences at 0.01 level of significance in Duncan test. White and black blocks on chromosomes represent genotypes of HJX74 and donor, respectively

Six SSSLs, A7, A13, A35, A37, A41 and A42 with significantly higher SER than HJX74, carried the substitution segments in the region of RM12521-RM12865 on chromosome 2 (Fig. 2a, c). A42, the SSSL with the longest of substitution segment, was then selected to develop secondary SSSLs. Six secondary SSSLs were developed from a F_2_ population derived from the cross of HJX74/A42 (Fig. 2b–d). The substitution segments of six secondary SSSLs were divided into two sub-set. In the left sub-set, the secondary SSSL A42-45 carrying the substitution segment from markers RM12521 to ID02M23 had low SER as HJX74, while secondary SSSLs A42-69 and A42-34 carrying longer substitution segments had significantly higher SER than HJX74. These results indicated that there was a QTL for SER, *qSER-2a*, locating in the region from markers ID02M23 to RM3732 with the estimated length of 234.9 kb. In the right sub-set, the secondary SSSL A42-12 carrying the substitution segment from markers ID02MQ21 to RM12865 had low SER as HJX74, while the SSSL A41 and the secondary SSSL A42-17 carrying longer substitution segments had significantly higher SER than HJX74. These results indicated that there was another QTL for SER, *qSER-2b*, locating in the region from markers ID02MQ51 to ID02MQ21 with the estimated length of 214.3 kb. The two QTLs for SER, *qSER-2a* and *qSER-2b*, were closely linked in the region of 1288.0 kb (Fig. 2d).

### Two closely linked QTLs for SER were dissected on the substitution segment of chromosome 3

**Fig. 3:**
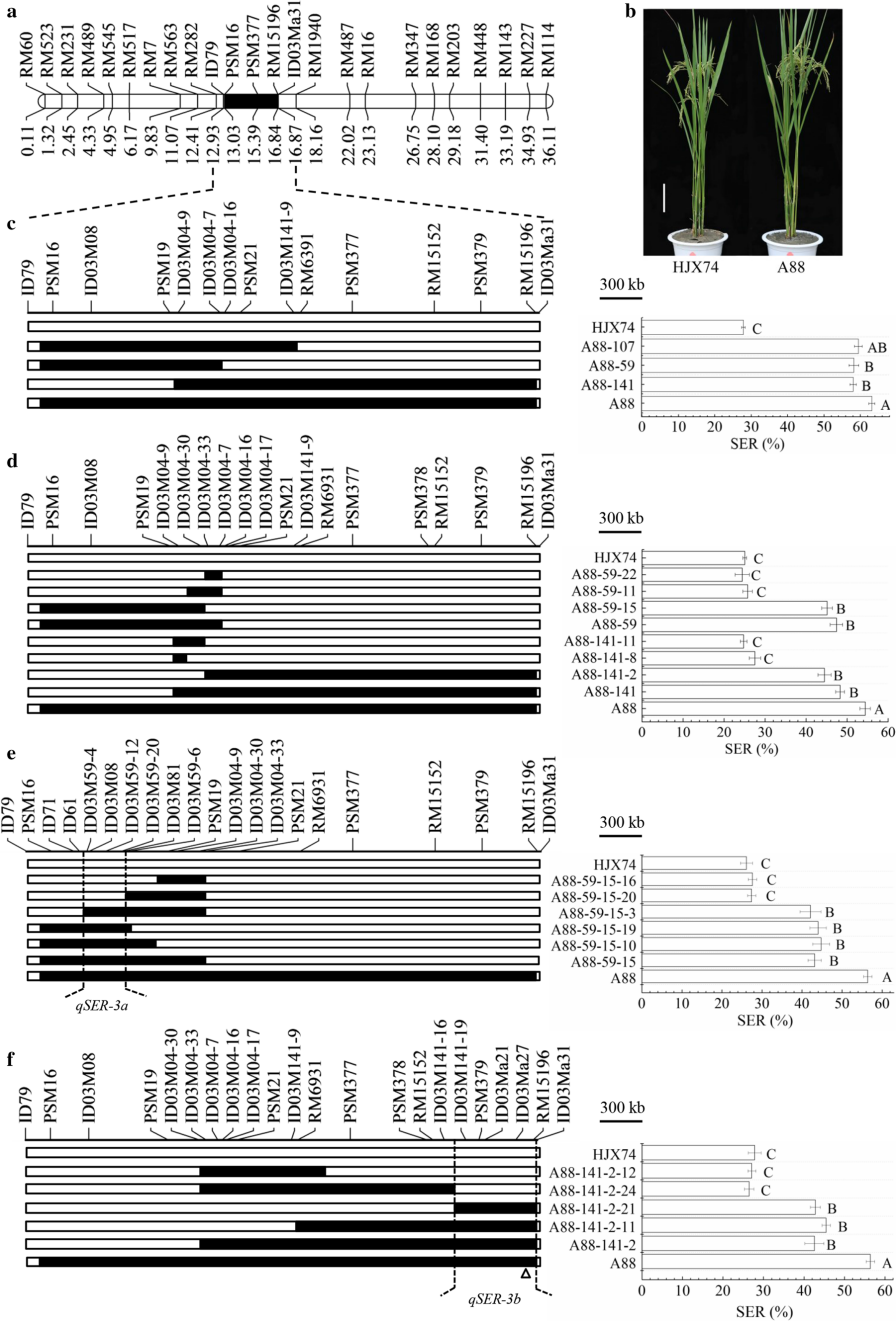
Substitution mapping of QTLs for SER on chromosome 3. **a** The substitution segment of SSSL-A88 on chromosome 3. Physical distance (Mb) was shown under the chromosome. **b** Plant type of HJX74 and SSSL-A88. Scale bar, 15 cm. **c** Secondary substitution mapping based on the substitution segment of SSSL-A88. **d** Secondary substitution mapping based on the substitution segment of A88-59 and A88-141, respectively. **e** Secondary substitution mapping of *qSER-3a* based on the substitution segment of A88-59-15. **f** Secondary substitution mapping of *qSER-3b* based on the substitution segment of A88-141-2. *SER* stigma exsertion rate. SER (%) was mean ± S.E. in two cropping seasons. Different alphabet letters denote differences at 0.01 level of significance in Duncan test. White and black blocks on chromosomes represent genotypes of HJX74 and donor, respectively. Δ, Location of *GS3*

The SSSL, A88, with significantly higher SER than HJX74, carried the substitution segment from markers PSM16 to RM15196 on chromosome 3 with the estimated length of 3874.7 kb (Fig. 3a–c and Table S3). A88 was used to develop secondary SSSLs. Three secondary SSSLs, A88-59, A88-107 and A88-141, were developed from the cross of A88/HJX74. The three secondary SSSLs carried different substitution segments showed significantly higher SER than HJX74 (Fig. 3c).

A88-59 and A88-141 were then used to develop secondary SSSLs. Three secondary SSSLs, A88-59-11, A88-59-15 and A88-59-22, were developed from the A88-59 heterozygote. Only A88-59-15 showed high SER as A88-59, while A88-59-11 and A88-59-22 had low SER as HJX74. Other three secondary SSSLs, A88-141-2, A88-141-8 and A88-141-11, were developed from the A88-141 heterozygote. Only A88-141-2 showed high SER as A88-141, while A88-141-8 and A88-141-11 had low SER as HJX74. Based on the substitution segments of the two sets of secondary SSSLs, the substitution segment of A88 should have two QTLs for SER. One was located in the interval from markers ID79 to ID03M04-9 on the left, and another was located in the interval from markers ID03M04-7 to ID03Ma31 on the right (Fig. 3d).

To narrow the QTL interval from markers ID79 to ID03M04-9 on the left, A88-59-15 was used to develop secondary SSSLs. Five secondary SSSLs, A88-59-15-3, A88-59-15-10, A88-59-15-16, A88-59-15-19 and A88-59-15-20, were developed from the A88-59-15 heterozygote. Three secondary SSSLs, A88-59-15-3, A88-59-15-10 and A88-59-15-19, showed high SER as A88-59-15, while two secondary SSSLs, A88-59-15-16 and A88-59-15-20, had low SER as HJX74. Therefore, the QTL, *qSER-3a*, was located in the interval from markers ID61 to ID03M81 with estimated length of 319.1 kb (Fig. 3e).

To narrow the QTL interval from markers ID03M04-7 to ID03Ma31 on the right, A88-141-2 were used to develop secondary SSSLs. Four secondary SSSLs, A88-141-2-11, A88-141-2-12, A88-141-2-21 and A88-141-2-24, were developed from the A88-141-2 heterozygote. A88-141-2-11 and A88-141-2-21 showed high SER as A88-141-2, while A88-141-2-12 and A88-141-2-24 had low SER as HJX74. Because A88-141-2-21 carried the substitution segment from markers ID03M141-16 to ID03Ma31, the QTL, *qSER-3b*, was located in the interval with estimated length of 637.3 kb (Fig. 3f).

The two QTLs for SER, *qSER-3a* and *qSER-3b*, were closely linked in the region of 3575.5 kb (Fig. 3e-f). We noted that the *GS3* locus controlling grain length was located at the right end of the *qSER-3b* interval between markers ID03Ma27 and RM15196. Genotyping of the *GS3* gene by a functional marker showed that A88 carried the *GS3* allele as same as HJX74 (Fig. S1). Phenotyping of A88 showed that the grain length was no significant difference with HJX74 (Table S4). These results indicated that the SER controlled by *qSER-3b* was not affected by the *GS3* gene.

### Additive and epistatic effects of the QTLs for SER

The two closely linked QTLs, *qSER-2a* and *qSER-2b*, were separated from the segment of chromosome 2. The secondary SSSLs only carrying *qSER-2a* or *qSER-2b* were used to evaluate the additive effects of the *qSER-2a* and *qSER-2b*, respectively (Table S5). The additive effects were 9.0% in *qSER-2a* and 8.8% in *qSER-2b* (Table 1). In the same way, the two closely linked QTLs, *qSER-3a* and *qSER-3b*, were dissected from the segment of chromosome 3. The sets of secondary SSSLs only carrying *qSER-3a* or *qSER-3b* were used to estimate the additive effects of the *qSER-3a* and *qSER-3b*, respectively (Table S6). The additive effects were 8.7% in *qSER-3a* and 7.9% in *qSER-3b* (Table 1).

**Table 1:** The additive effects of the QTLs for stigma exsertion rate detected in the SSSLs

QTL	Chr	Interval (kb)	Estimated length (kb)	Maximum length (kb)	Additive effect (%)
*qSER-2a*	2	4121.8–4356.8	234.9	370.7	9.0 ± 0.3
*qSER-2b*	2	5195.5–5409.8	214.3	291.5	8.8 ± 0.7
*qSER-3a*	3	13,277.6–13,596.7	319.1	361.4	8.7 ± 0.3
*qSER-3b*	3	16,215.9–16,853.1	637.3	736.3	7.9 ± 0.5

In the same experiments, the four lines carrying the substitution segments with both *qSER-2a* and *qSER-2b*, A35, A37, A42 and A42-24, showed significantly higher SER than the lines only with *qSER-2a* or *qSER-2b* (Table S5 and Fig. 2d). Similarly, the SSSL A88 carrying both *qSER-3a* and *qSER-3b* had significantly higher SER than those only carrying *qSER-3a* or *qSER-3b* (Table S6 and Fig. 3d-f). These results indicated that the two pairs of QTLs had significantly additive effects.

**Fig. 4:**
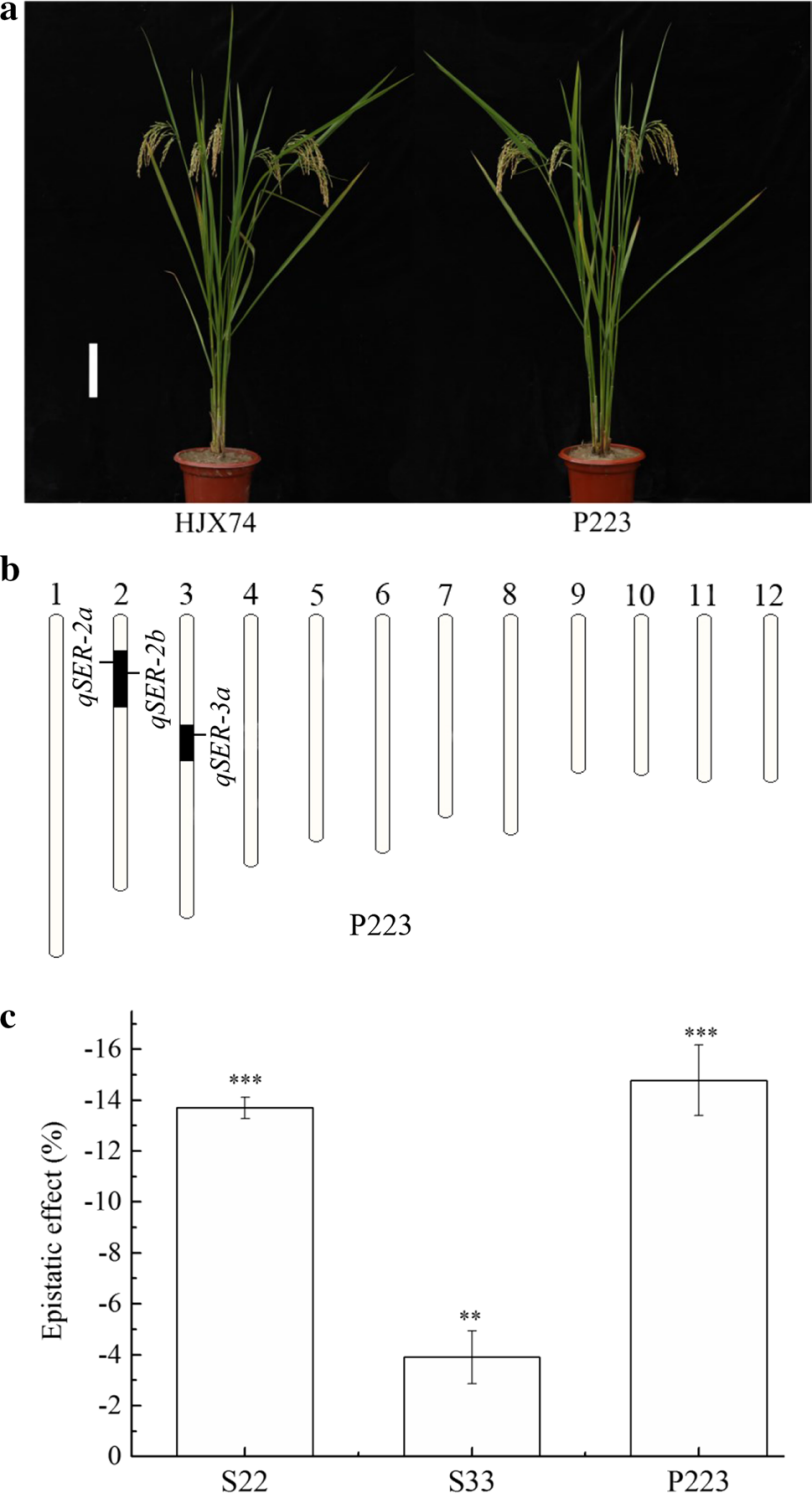
Epistatic effects estimated in the lines with two or three QTLs for SER. **a** Plant type of HJX74 and pyramiding line P223. Scale bar, 15 cm. **b** Position of the three QTLs for SER in P223. Black part represents substitution segments from IR66897B (IB) with target QTLs, and white part is the genetic background of HJX74. **c** Epistatic effects estimated in S22, S33 and P223. S22 is a group of the SSSLs carrying *qSER-2a* and *qSER-2b*. S33 is a group of the SSSLs carrying *qSER-3a* and *qSER-3b*. P223 is a pyramiding line with *qSER-2a*, *qSER-2b* and *qSER-3a*. Student’s *t* test. *SER* stigma exsertion rate, *SSSL* single-segment substitution line

To evaluate epistatic effects produced by the interaction of QTLs for SER, the SSSL A42 having *qSER-2a* and *qSER-2b* and the secondary SSSL A88-59-15 with *qSER-3a* were used to develop the pyramiding line P223 carrying *qSER-2a*, *qSER-2b* and *qSER-3a* (Fig. 4a, b). In single-QTL groups of SSSLs, S2a having *qSER-2a*, S2b having *qSER-2b*, S3a having *qSER-3a* and S3b having *qSER-3b*, showed SER of 46.3%, 46.0%, 43.5% and 43.7%, respectively, while two-QTL groups showed 51.3% SER in S22 carrying *qSER-2a* and *qSER-2b*, and 56.3% SER in S33 of *qSER-3a* and *qSER-3b*. SER of the two-QTL groups was significantly higher than those of the single-QTL groups (Table S7). Based on the SER data, the additive effect of the pair of *qSER-3a* and *qSER-3b* in S33 group was 14.6%, which was much greater than 12.2% of the *qSER-2a* and *qSER-2b* pair in S22 group. This was because the epistatic effect produced by the interaction of *qSER-2a* and *qSER-2b* was much greater than that of *qSER-3a* and *qSER-3b*, with the former being -13.9% and the latter being -3.9% (Fig. 4c). As a comparison, SER of the pyramiding line P223 with three QTLs was 67.2%, greatly higher than those of single-QTL groups and of two-QTL groups (Table S7). The epistatic effect in P223 was -14.6%, which seems mainly from the interaction of *qSER-2a* and *qSER-2b* (Fig. 4c).

### Gene annotation in the regions of *qSER-2a*, *qSER-2b* and *qSER-3a*

The ORFs within the maximum intervals of three fine mapped QTLs for SER were identified. From the RAP-DB and the MSU-RAP, 64 and 56 of ORFs were, respectively, identified within 370.7 kb of the *qSER-2a* interval. Among them, 45 ORFs were identified from the two databases. In the 291.5 kb of *qSER-2b* region, 51 and 45 of ORFs were, respectively, identified from the RAP-DB and the MSU-RAP and 36 of them were identified from the two databases. The *qSER-3a* region with 361.4 kb included 19 common ORFs, which from 22 and 51 ones identified from the RAP-DB and the MSU-RAP (Fig. S2).

In the regions of three QTLs, most identifiers only from the RAP-DB were hypothetical genes or non-protein coding transcripts, and most identifiers only from the MSU-RAP were hypothetical genes, retrotransposon genes or transposon genes (Tables S8-S10). Therefore, the common identifiers from both databases are more likely to be candidate genes of the QTLs.

## Discussion

Many QTLs for SER and related traits have been identified and are distributed across all the 12 chromosomes. However, few of the QTLs were located within a 500 kb interval (Marathi and Jena 2015; Liu et al. 2019; Tan et al. 2020). In the present study, we detected two QTLs for SER in the region of 1288.0 kb on chromosome 2. *qSER-2a* was mapped in the estimated interval of 234.9 kb, and *qSER-2b* was located in 214.3 kb estimated interval. In previous studies, five QTLs for SER were detected around the region. Li et al. (2003) detected a QTL, *qPES-2*, in the interval of 1044 kb using a DH population. Deng et al. (2010) located *qPES-2* in the region of about 770 kb using an F_2_ population. Li et al. (2017) mapped *qPES2.2* in 4993 kb region using an F_2: 3_ population. Zou et al. (2020) identified *qSERr2-1* in about 3221 kb region from *O. rufipogon* and *qSERm2-1* in the region of about 6739 kb from *O*. *meridionalis*. On chromosome 3, we detected two QTLs for SER in the region of 3575.5 kb. *qSER-3a* was mapped in the estimated interval of 319.1 kb, and *qSER-3b* was located in 637.3 kb estimated interval. In previous studies, seven QTLs for SER were detected around the region. Miyata et al. (2007) detected a QTL, *qES3*, in the interval of 10554 kb using a CSSL population. Yue et al. (2009) located *PES-3* in 7895 kb region using a F_2_ population. Feng et al. (2010) mapped *qSPE3* in the region of about 600 kb using a F_2_ population. Li et al. (2014) identified *qSSE3* in 14426 kb interval using a RIL population. Tan et al. (2020) detected *qSER-3a* in the region of 4632 kb from *O. glumaepatula*. Zou et al. (2020) identified *qSERb3-1* in the region of 6629 kb from *O. barthii* and *qSERm3-1* in the 2983 kb region from *O*. *meridionalis*. These results revealed that there are potential QTL clusters for SER in the two regions of chromosome 2 and chromosome 3. From the RAP-DB and the MSU-RAP, the open reading frames (ORFs) were identified within the maximum intervals of *qSER-2a*, *qSER-2b* and *qSER-3a*, respectively. The dissection of two pairs of closely linked QTLs for SER and the fine mapping of the QTLs laid a foundation for the cloning of genes for SER.

During the process of domestication, cultivated rice has already lost some traits of natural outcrossing (Parmar et al. 1979). Wild *Oryza* species have a strong outcrossing ability due to their larger stigma, longer style, greater exsertion of the stigma, and longer periods of spikelet opening (Marathi et al. 2015; Marathi and Jena 2015). It was found that cultivated rice tends to have a shorter stigma than the annual wild species, while annual wild species have shorter stigma than their perennial progenitors (Oka and Morishima 1967; Virmani and Athwal 1973; Parmar et al. 1979; Marathi et al. 2015). Previous studies revealed dominant differences for SER and floral traits between cultivated rice and wild rice (Virmani and Athwal 1973; Uga et al. 2003b). Recently, seven QTLs for SER from *O. glumaepatula*, a wild *Oryza* species, were located on five chromosomes by substitution mapping. The additive effects of seven QTLs ranged from 10.6 to 14.8% (Tan et al. 2020). In the present study, four QTLs for SER identified in cultivated rice had their additive effects from 7.9 to 9.0% (Table 1). Obviously, the additive effect of the QTLs for SER in cultivated rice was usually lower than that in wild rice. This may be one of the reasons for the decrease of outcrossing ability of cultivated rice during domestication.

In order to detect QTLs of rice by substitution mapping, we constructed a library containing 2360 SSSLs using 43 accessions from seven species of *Oryza* AA-genome as donors and Huajingxian74 (HJX74), an elite *indica* variety, as the recipient (Zhang et al. 2004; Xi et al. 2006; He et al. 2017; Zhao et al. 2019; Zhang 2019). Similar to NILs, each SSSL carries only one chromosomal segment from a donor under the genetic background of recipient (Zhang et al. 2004; Xi et al. 2006). Since the SSSLs have homozygous genotypes, the SSSLs can be used to test the phenotype in different cropping seasons. This increases the accuracy of phenotyping of complex traits (Tan et al. 2020). Because the QTLs for SER had minor effects and were closely linked, the QTLs were not easy to be dissected using traditional mapping methods. As mentioned above, we dissected two closely linked QTLs in the QTL cluster region of chromosomes 2 and 3, whereas only one QTL was detected in each previous study. These facts indicated that substitution mapping was a powerful tool for dissection of closely linked QTLs of complex traits.

Based on the HJX74-SSSL library, the platform of breeding by design in rice has been developed (Dai et al. 2015, 2016; Luan et al. 2019; Zhang 2019). In this study, we found that the *qSER-2a* and *qSER-2b* on chromosome 2 and the *qSER-3a* and *qSER-3b* on chromosome 3 had significant additive effects for SER. In comparison, the epistatic effect produced by the interaction of *qSER-2a* and *qSER-2b* was much greater than that of *qSER-3a* and *qSER-3b*. Therefore, the SSSLs carrying two closely linked QTLs, *qSER-2a* and *qSER-2b* on chromosome 2 or *qSER-3a* and *qSER-3b* on chromosome 3, will be favorable genetic resources for developing MSLs with high SER. Understanding the epistatic effect of QTL interaction is important for breeding by design.

## Supplementary information

Below is the link to the electronic supplementary material.

Supplementary material 1 (PDF 506 kb)
