# Supplementary material for: Dissection of closely linked QTLs controlling stigma exsertion rate in rice by substitution mapping
Source: Theor Appl Genet. 2021 Jan 25;134(4):1253–62. doi: 10.1007/s00122-021-03771-9 (PMC7973394; doi:10.1007/s00122-021-03771-9)
Supplement: Supplementary file 1 — Supplementary material 1 (PDF 506 kb) [file 122_2021_3771_MOESM1_ESM.pdf]

Table S1 Stigma exsertion rate of the 7 SSSLs in 9 cropping seasons from 2014 to 2018

SSSL	Chr.	Donor	SER (%)										*P* value of SER									
			2014 FCS	2014 SCS	2015 FCS	2015 SCS	2016 FCS	2016 SCS	2017 FCS	2017 SCS	2018 FCS	Average	2014 FCS	2014 SCS	2015 FCS	2015 SCS	2016 FCS	2016 SCS	2017 FCS	2017 SCS	2018 FCS	Average
A88	3	IB	49.69±2.25	43.00±1.82	52.47±2.10	61.17±1.93	50.35±1.67	62.82±1.09	56.64±1.53	63.62±3.75	57.89±1.63	55.37±1.35	1.34E-09	1.34E-09	1.34E-09	1.44E-08	1.34E-09	1.00E-05	3.10E-08	8.05E-08	1.94E-08	2.23E-08
A7	2	IB	40.88±1.96	34.84±1.22	49.93±1.82	44.02±0.80	40.16±1.14	47.48±0.89	47.82±1.79	55.39±0.97	50.05±0.82	45.63±1.19	2.39E-09	5.35E-08	1.34E-09	2.79E-05	1.59E-09	1.00E-04	8.02E-08	2.16E-06	1.94E-08	2.23E-08
A13	2	IB	37.31±2.01	46.17±2.25	45.11±2.35	60.44±1.03	45.62±1.60	52.66±0.74	56.57±0.85	63.62±2.01	48.87±1.97	50.73±1.58	4.82E-07	1.34E-09	1.73E-09	1.44E-08	1.34E-09	1.00E-05	3.10E-08	8.16E-08	1.94E-08	2.23E-08
A35	2	IB	39.20±1.25	34.91±1.77	43.89±2.17	55.39±2.19	41.67±1.30	40.64±1.13	44.78±1.54	56.59±3.05	46.05±1.76	44.82±1.39	1.93E-08	4.97E-08	3.89E-09	1.44E-08	1.35E-09	1.59E-04	4.52E-07	1.21E-06	1.94E-08	2.23E-08
A37	2	IB	39.44±2.01	38.31±1.44	45.29±1.60	53.34±2.04	40.76±1.55	41.10±0.82	42.29±1.67	56.13±1.78	46.10±0.86	44.74±1.21	1.42E-08	1.44E-09	1.62E-09	1.44E-08	1.40E-09	5.00E-04	3.05E-06	1.51E-06	1.94E-08	2.23E-08
A41	2	IB	34.11±0.97	35.98±1.78	39.21±1.69	51.65±1.74	39.92±0.99	51.02±1.24	41.59±0.84	62.97±0.69	47.89±1.21	45.08±1.72	7.88E-05	8.55E-09	3.06E-06	1.45E-08	1.77E-09	1.00E-05	5.44E-06	9.81E-08	1.94E-08	2.23E-08
A42	2	IB	38.84±1.79	40.40±1.56	44.23±2.06	59.84±1.30	45.87±1.07	62.20±1.42	50.61±1.04	65.82±2.15	52.66±2.14	50.93±1.88	3.73E-08	1.34E-09	2.83E-09	1.44E-08	1.34E-09	1.00E-05	3.91E-08	5.12E-08	1.94E-08	2.23E-08
HJX74 (CK)	-	-	23.40±1.16	20.49±1.43	24.84±1.50	33.48±0.23	26.66±0.64	35.37±0.90	27.34±1.22	29.28±1.52	29.31±0.40	27.83±0.91										

SER stigma exsertion rate, *IB* IR66897B, *HJX74* as control, *FCS* the first cropping season, *SCS* the second cropping season. Values were mean ± S.E. The *P* value were from Dunnett *t*-test between each SSSL and HJX74.

Table S2 Markers developed for detection of the substituted segments of SSSLs

Primer name	Forward primer	Reverse primer
ID79	ACGTACACACAGCACACAAT	CAAAAAGGTTAATTTGCTTG
PSM16	GGTCTTAATGCCCTCTTCCC	ACAACCAAACAAGCCATCCT
ID71	GATGAACTCCACCACGAA	GAGAAGGGGAGGAGAGTG
ID61	GTTCTGGTTGGTAGGTTTCA	TTGGGAGCTTTTCTTCTGTA
ID03M59-4	TAAAACAGCATACCATGCAA	ATTAAACTTGCAGTTGGCTC
ID03M08	TAGCGGTTAAGGAAAGACAG	CCAAGAGAGCTAGACTTCCA
ID03M59-12	GTGGACCGAGAGGCTGAC	GGTATGGCCATTTGAAGTT
ID03M59-20	GTGGATGTCGGTCTCCAAGT	AGGTTTGAGGCAGGGGTAAT
ID03M81	TGAAAGGAATAAAAATCTAA	TTCTAACGGGAGGATTTATG
ID03M59-6	GACGATAAAATCTTGTCCCA	TCTGTTTACGTTCTCGGTTT
PSM19	CGAGTAAGGGTTCACCAATCA	GCATCGGTCTCCTCCGCAGC
ID03M04-9	TAGATCAGGCTGAGTCCATT	TGGCAAGTACAATCACAAAA
ID03M04-30	ACCTGAACAAACGGAGTAGA	GAGACGTCACAATCCTCTTC
ID03M04-33	CCTGGGACGATCAATTTT	TTCGAATTTGAATAGGGTAT
ID03M04-7	TGTTATAACCTACAGGCCAG	GGTACGTCTTGCTCAGGAAC
ID03M04-16	AAATAGCAGTGCGTTGAAAT	CAGCTGATAATATGGATGGC
ID03M04-17	GTGTATTCCCATCATCGTGT	ATTAAGTTCCTCTGTGCTCC
PSM21	CAGCATTATTCTGGTTGGCATTT	ATACATCGGCGGAGGAGCA
ID03M141-9	ACAGCTACAGGGTATGTCGT	TCTCAATTTGTAAGGTTGCA
PSM377	CTCACAAGAACGCCACAAAA	TGACCAGAAAGGAGAAGGAAA
PSM378	TGCTTATTGGTTTGTCTTGGC	AAGAATAGCCTTTGCCTCCA
ID03M141-16	AGTTGTATCTGCCTCCAAAA	CTTCTACCTCAGGAGCTTCA
ID03M141-19	AGGACAAGTGTTTCCAAGAA	ATTGCCACAGTTCAACCTAC
PSM379	GCATCTCCTTTTGCTGTTTCC	TGCCTCGTCTCCATCAAGTTTT
ID03Ma21	CAACCATATTGGCCTATCTA	GAGCTTGGTGTCTATAACCG
ID03Ma27	AGACACACATAGTGGGCATT	AGAAAAGGTCTGGATAGATG
ID03Ma31	ATATGCAGATCAATCAACGG	ACCAGCAAATGACTAAATTG
ID02M23	ATGCGCTTTATGGGTACTTA	TTTGCTTCTGGTAAACACCT
PSM116	GTCCCCTCTCCTCCATTCTC	CCCCCTCTCCTCACAATTC
ID02MQ51	CGCTACTTTTCTCTCTCCTG	ACTGACTCGGGAGAATACTG
PSM117	GGTTGTGCCATGGCTAGTTT	CGCGAGCGTTTTAGCATAAT
ID02MQ21	GATCATCTCATGCCTCCTAC	CATCTCCCAATATGACCAAC
PSM119	CAGAGAGAGAGCCCAGAGGA	TCTGTGCTAGCAGCCTCTCA
ID02M01	CATAGCCTAGTCGGAAATGA	CGTTAACGATGGAGGTTAAG
ID02M04	ATCACGTCACCTTGCATATC	CTACTACTCTTGGCCGTGAC

Table S3 Substituted segments of the SSSLs

SSSL	Chr.	Donor	QTL	Substituted segment	Position of chromosomes (kb)				Length of segments (kb)		
					P1	P2	P3	P4	L_{min}	L_{max}	L_{est}
A88	3	IB	*qSER-3a* , *qSER-3b*	ID79--PSM16-ID71-ID61-ID03M59-4-ID03M08-ID03M59-12-ID03M59-20-ID03M81-ID03M59-6-PSM19-ID03M04-9-ID03M04-30-ID03M04-33-ID03M04-7-ID03M04-16-ID03M04-17-PSM21-ID03M141-9-RM6931-PSM377-PSM378-RM15152-ID03M141-16-ID03M141-19-PSM379-ID03Ma21-ID03Ma27-RM15196--ID03Ma31	12930.12	13026.79	16838.82	16867.46	3812.03	3937.34	3874.68
A7	2	IB	*qSER-2a* , *qSER-2b*	RM7652--RM12521-RM1285-ID02M23-RM7581-RM5622-RM555-RM3732-PSM116-ID02MQ51-PSM117-RM12655-ID02MQ21-PSM119-RM5897--RM492	3478.72	3653.93	6733.54	7285.64	3079.61	3806.92	3443.26
A13	2	IB	*qSER-2a* , *qSER-2b*	RM7652--RM12521-RM1285-ID02M23-RM7581-RM5622-RM555-RM3732-PSM116-ID02MQ51-PSM117-RM12655-ID02MQ21-PSM119-RM5897-RM492-RM322-RM145-ID02M01--ID02M04	3478.72	3653.93	7850.76	8359.48	4196.84	4880.76	4538.80
A35	2	IB	*qSER-2a* , *qSER-2b*	RM7652--RM12521-RM1285-ID02M23-RM7581-RM5622-RM555-RM3732-PSM116-ID02MQ51-PSM117-RM12655--ID02MQ21	3478.72	3653.93	5341.99	5477.52	1688.06	1998.80	1843.43
A37	2	IB	*qSER-2a* , *qSER-2b*	ID02M23--RM7581-RM5622-RM555-RM3732-PSM116-ID02MQ51-PSM117-RM12655-ID02MQ21-PSM119-RM5897-RM492-RM322-RM145-ID02M01-ID02M04-RM12855-RM12865--RM71	4037.21	4206.48	8697.37	8760.43	4490.89	4723.23	4607.06
A41	2	IB	*qSER-2b*	ID02MQ51--PSM117-RM12655-ID02MQ21-PSM119-RM5897-RM492-RM322-RM145-ID02M01-ID02M04-RM12855-RM12865--RM71	5186.03	5204.96	8697.37	8760.43	3492.41	3574.41	3533.41
A42	2	IB	*qSER-2a* , *qSER-2b*	RM7652--RM12521-RM1285-ID02M23-RM7581-RM5622-RM555-RM3732-PSM116-ID02MQ51-PSM117-RM12655-ID02MQ21-PSM119-RM5897-RM492-RM322-RM145-ID02M01-ID02M04-RM12855-RM12865--RM71	3478.72	3653.93	8697.37	8760.43	5043.44	5281.71	5162.58

IB IR66897B, P2 and P3 are the marker positions of donor genotype at the front-end and after-end of the substitution segment, respectively. P1 and P4 are the marker positions of background genotype flanking out of the front-end and after-end of the substitution segment, respectively. L_{min} = minimum length of substituted segment (kb) = P3 - P2, L_{max} = maximum length of substituted segment (kb) = P4 - P1, L_{est} = estimated length of substituted segment (kb) = (L_{min} + L_{max}) / 2. '--' represents the region of chromosome recombination. '-' represents connection of substituted segment.

Table S4 Phenotypes of agronomic traits in the 7 SSSLs and HJX74

SSSL	Heading date (d)		Plant height (cm)		Grain number per panicle		Panicle length (cm)		Seed setting rate (%)		Grain length (mm)		Grain width (mm)		Thousand grain weight (g)	
	2018 FCS	2018 SCS	2018 FCS	2018 SCS	2018 FCS	2018 SCS	2018 FCS	2018 SCS	2018 FCS	2018 SCS	2018 FCS	2018 SCS	2018 FCS	2018 SCS	2018 FCS	2018 SCS
A88	97.80±0.42	73.78±0.40	107.30±0.96	95.10±0.85	222.19±11.50	183.15±7.22*	23.76±0.24	21.46±0.24*	74.20±1.93	84.00±0.77	8.24±0.03	8.44±0.03	2.63±0.01	2.61±0.02	21.19±0.21	23.01±0.40
A7	96.50±0.50*	73.00±0.47	110.10±0.64	93.30±0.76*	211.92±7.23*	203.28±3.55	22.58±0.24**	23.07±0.33	78.00±1.70	85.20±0.97	8.17±0.02	8.34±0.02	2.65±0.02	2.57±0.01	21.17±0.36	21.45±0.07**
A13	96.30±0.37**	73.10±0.46	110.00±1.74	95.30±0.47	253.22±8.92	181.02±12.16	25.14±0.34	24.32±0.34	77.80±2.75	83.60±0.98	8.31±0.03	8.50±0.04	2.56±0.02**	2.54±0.01*	20.55±0.25	22.09±0.34*
A35	97.30±0.47	73.10±0.48	112.40±0.91**	98.30±0.88	224.63±16.18	219.51±7.76	22.71±0.38**	23.13±0.28	77.40±2.29	87.00±1.14*	8.24±0.03	8.43±0.04	2.57±0.01**	2.57±0.02	20.16±0.40*	21.74±0.24**
A37	96.70±0.52*	72.30±0.54**	112.20±0.73**	97.80±1.12	231.27±13.32	220.17±12.50	23.46±0.42*	22.79±0.33	77.80±2.08	86.80±0.49	8.22±0.02	8.36±0.04	2.67±0.02	2.61±0.01	20.89±0.34	22.71±0.16
A41	97.70±0.47	76.00±0.33	109.20±0.99	97.50±1.15	213.76±7.81*	206.57±7.18	22.56±0.30**	22.71±0.71	76.40±1.69	84.40±0.68	8.37±0.02	8.41±0.04	2.60±0.01	2.58±0.02	21.34±0.16	22.62±0.20
A42	97.10±0.43	73.10±0.64	109.60±0.79	96.70±1.26	212.63±18.90*	180.42±6.12*	23.35±0.69*	22.24±0.29	77.40±2.50	81.00±2.81	8.33±0.02	8.42±0.02	2.58±0.01**	2.57±0.01	20.47±0.28	21.76±0.22**
HJX74	98.60±0.60	74.98±0.45	107.00±1.02	96.23±0.42	258.44±8.58	209.97±5.89	24.81±0.22	23.16±0.33	74.70±1.62	82.40±0.87	8.26±0.03	8.45±0.03	2.64±0.01	2.60±0.01	21.15±0.19	23.06±0.14

HJX74 as control, *FCS* the first cropping season, *SCS* the second cropping season. Data were presented as mean ± S.E., * P -value ≤ 0.05, ** P -value ≤ 0.01, Dunnett t-test.

Table S5 The stigma exsertion rate of the SSSLs and secondary SSSLs used to map *qSER-2a* or/and *qSER-2b*

Secondary SSSL	QTL	SER (%)		
		2018 SCS	2019 FCS	Average
A42-34	*qSER-2a*	45.83±1.45^{B}	47.66±2.59^{AB}	46.75±1.24^{B}
A42-69	*qSER-2a*	44.53±1.94^{B}	47.06±1.75^{AB}	45.79±1.51^{B}
A42-17	*qSER-2b*	46.41±1.38^{B}	48.22±1.20^{AB}	47.32±0.69^{B}
A41	*qSER-2b*	44.14±1.30^{B}	45.27±1.85^{B}	44.71±1.82^{B}
A42-24	*qSER-2a* , *qSER-2b*	51.12±2.57^{A}	51.58±2.16^{AB}	51.35±1.01^{A}
A35	*qSER-2a* , *qSER-2b*	50.78±1.26^{A}	51.85±1.12^{AB}	51.32±3.04^{A}
A37	*qSER-2a* , *qSER-2b*	50.42±1.43^{A}	52.05±2.07^{A}	51.23±2.90^{A}
A42	*qSER-2a* , *qSER-2b*	51.31±2.68^{A}	51.64±1.58^{AB}	51.47±0.59^{A}
A42-45	-	27.62±0.73^{C}	27.92±1.32^{C}	27.77±0.68^{C}
A42-12	-	28.91±0.99^{C}	27.74±1.15^{C}	28.32±0.73^{C}
HJX74 (Control)	-	27.66±0.41^{C}	28.96±0.53^{C}	28.31±0.57^{C}

FCS the first cropping season, *SCS* the second cropping season. Data were presented as mean ± S.E., α =0.01, Duncan. Average were calculated from values of all samples in two cropping seasons.

Table S6 The stigma exsertion rate of the SSSL and secondary SSSLs used to map *qSER-3a* or/and *qSER-3b*

Secondary SSSL	QTL	SER (%)		
		2018 FCS	2018 SCS	Average
qSER-3a				
A88-59-15-3	*qSER-3a*	41.52 ± 1.93^{B}	42.67 ± 2.02^{B}	42.10 ± 2.63^{B}
A88-59-15-19	*qSER-3a*	42.55 ± 1.35^{B}	45.61 ± 1.87^{B}	44.08 ± 2.03^{B}
A88-59-15-10	*qSER-3a*	43.98 ± 2.08^{B}	45.52 ± 1.84^{B}	44.75 ± 2.13^{B}
A88-59-15	*qSER-3a*	42.40 ± 0.83^{B}	43.85 ± 1.44^{B}	43.13 ± 1.69^{B}
A88	*qSER-3a* , *qSER-3b*	57.89 ± 1.62^{A}	54.61 ± 0.72^{A}	56.40 ± 1.04^{A}
A88-59-15-16	-	26.27 ± 0.90^{C}	28.91 ± 1.52^{C}	27.59 ± 1.07^{C}
A88-59-15-20	-	27.41 ± 0.88^{C}	27.40 ± 1.18^{C}	27.41 ± 1.03^{C}
HJX74 (Control)	-	25.97 ± 1.64^{C}	26.23 ± 0.59^{C}	26.10 ± 1.50^{C}
qSER-3b				
A88-141-2	*qSER-3b*	42.25 ± 2.00^{B}	42.84 ± 1.72^{B}	42.54 ± 2.33^{B}
A88-141-2-11	*qSER-3b*	44.95 ± 0.94^{B}	46.41 ± 1.57^{B}	45.68 ± 1.02^{B}
A88-141-2-21	*qSER-3b*	42.13 ± 0.92^{B}	43.49 ± 1.11^{B}	42.81 ± 1.25^{B}
A88	*qSER-3a* , *qSER-3b*	57.89 ± 1.62^{A}	54.61 ± 0.72^{A}	56.40 ± 1.04^{A}
A88-141-2-24	-	26.23 ± 0.73^{C}	26.70 ± 1.87^{C}	26.46 ± 1.13^{C}
A88-141-2-12	-	26.50 ± 0.79^{C}	27.77 ± 0.93^{C}	27.13 ± 0.99^{C}
HJX74 (Control)	-	28.40 ± 0.89^{C}	27.18 ± 1.30^{C}	27.79 ± 1.57^{C}

FCS the first cropping season, *SCS* the second cropping season. Data were presented as mean ± S.E., α=0.01, Duncan. Average were calculated from values of all samples in two cropping seasons.

Table S7 Comparison of SER, additive effects and epistatic effects in the pyramiding line P223 with those in single-QTL groups and two-QTL groups of SSSLs

Group	Genotype	SER (%)	Additive effect (%)	Epistatic effect (%)
S2a	*qSER-2a*	$46.27±0.71^{D}$	9.62±0.36***	-
S2b	*qSER-2b*	$46.01±1.32^{D}$	9.49±0.66***	-
S3a	*qSER-3a*	$43.51±0.59^{D}$	8.24±0.30***	-
S3b	*qSER-3b*	$43.68±1.01^{D}$	8.32±0.50***	-
S22	*qSER-2a* , *qSER-2b*	$51.34±0.42^{C}$	12.15±0.21***	-13.90±0.42***
S33	*qSER-3a* , *qSER-3b*	$56.25±1.04^{B}$	14.61±0.52***	-3.90±1.04**
P223	*qSER-2a* , *qSER-2b* , *qSER-3a*	$67.18±1.37^{A}$	20.07±0.69***	-14.58±1.38***
HJX74	Control	$27.04±0.62^{E}$	-	-

SER , stigma exsertion rate. Data were presented as mean ± S.E.. The SER data is averages of those from 2018 FCS to 2019 FCS. Differences of SER was tested by the Duncan, α = 0.01. Significances of additive effect and epistatic effect were tested by the Student's *t* test respectively. "**" and "***" indicated the significance at 0.01 and 0.001 level, respectively. *FCS* the first cropping season, *SCS* the second cropping season.

Table S8 The gene annotation within the maximum interval of *qSER-2a* identified from the databases of RAP and MSU

RAP-ID	MSU-ID	Gene annotation
Os02g0173700	LOC_Os02g07740	RNA binding protein, putative, expressed
Os02g0173800	LOC_Os02g07750	Cell cycle control protein, putative, expressed
Os02g0173900	LOC_Os02g07760	Aldehyde dehydrogenase, putative, expressed
Os02g0174000	LOC_Os02g07770	Myb transcription factor, putative, expressed
Os02g0174100	LOC_Os02g07780	OsSPL4-SBP-box gene family member, expressed
Os02g0174200	LOC_Os02g07790	Serine/threonine-protein kinase HT1, putative, expressed
Os02g0174300	LOC_Os02g07800	Transcription factor, putative, expressed
Os02g0174400	LOC_Os02g07810	Inactive receptor kinase At2g26730 precursor, putative, expressed
Os02g0174800	LOC_Os02g07820	DUF581 domain containing protein, expressed
Os02g0175000	LOC_Os02g07830	OsHKT1;3 - Na+ transporter, expressed
Os02g0175100	LOC_Os02g07840	BZIP transcription factor domain containing protein, expressed
Os02g0175302	LOC_Os02g07860	Conserved hypothetical protein
Os02g0175400	LOC_Os02g07870	ATP synthase, putative, expressed
Os02g0175500	LOC_Os02g07880	DNA binding protein, putative, expressed
Os02g0175600	LOC_Os02g07890	60S ribosomal protein L27a-3, putative, expressed
Os02g0175700	LOC_Os02g07900	ANTH/ENTH domain containing protein, putative, expressed
Os02g0175800	LOC_Os02g07910	Fiber protein Fb15, putative, expressed
Os02g0175900	LOC_Os02g07920	Expressed protein
Os02g0176000	LOC_Os02g07930	B-box zinc finger family protein, putative, expressed
Os02g0176100	LOC_Os02g07960	STRUBBELIG-RECEPTOR FAMILY 3 precursor, putative, expressed
Os02g0176200	LOC_Os02g07970	Expressed protein
Os02g0176400	LOC_Os02g07980	Expressed protein
Os02g0176600	LOC_Os02g08000	Expressed protein
Os02g0176650	LOC_Os02g08010	LTPL43-Protease inhibitor/seed storage/LTP family protein precursor, putative, expressed
Os02g0176700	LOC_Os02g08014	Calcium-transporting ATPase 9, plasma membrane-type, putative, expressed
Os02g0176900	LOC_Os02g08030	Aldose 1-epimerase, putative, expressed
Os02g0177300	LOC_Os02g08070	OsSPL5-SBP-box gene family member, expressed
Os02g0177400	LOC_Os02g08080	Expressed protein
Os02g0177500	LOC_Os02g08090	LSM domain containing protein, expressed
Os02g0177600	LOC_Os02g08100	AMP-binding domain containing protein, expressed
Os02g0177700	LOC_Os02g08110	Membrane associated DUF588 domain containing protein, putative, expressed
Os02g0177800	LOC_Os02g08120	Calmodulin binding protein, putative, expressed
Os02g0177900	LOC_Os02g08130	FGGY family of carbohydrate kinases, putative, expressed
Os02g0178000	LOC_Os02g08140	CAMK_KIN1/SNF1/Nim1_like.14-CAMK includes calcium/calmodulin depedent protein kinases, expressed
Os02g0178100	LOC_Os02g08150	CCT/B-box zinc finger protein, putative, expressed
Os02g0178200	LOC_Os02g08160	OsFBD6-F-box and FBD domain containing protein, expressed
Os02g0178300	LOC_Os02g08170	GDU1, putative, expressed
Os02g0178400	LOC_Os02g08180	Protein transport protein SEC61 subunit gamma, putative, expressed
Os02g0178500	LOC_Os02g08190	CYPRO4, putative, expressed
Os02g0178600	LOC_Os02g08200	Zinc finger, C3HC4 type domain containing protein, expressed
Os02g0178700	LOC_Os02g08220	Expressed protein
Os02g0178800	LOC_Os02g08230	WAX2, putative, expressed
Os02g0179000	LOC_Os02g08250	Expressed protein
Os02g0179100	LOC_Os02g08260	HD domain containing protein 2, putative, expressed
Os02g0179200	LOC_Os02g08270	Class I glutamine amidotransferase, putative, expressed
Os02g0174250		Hypothetical protein
Os02g0174450		Hypothetical gene
Os02g0174500		Hypothetical gene
Os02g0174700		Hypothetical protein
Os02g0174900		Hypothetical gene
Os02g0175200		DUF630/DUF632 domains containing protein, putative, expressed
Os02g0175250		Hypothetical protein
Os02g0175301		DUF630/DUF632 domains containing protein, putative, expressed
Os02g0175750		Hypothetical protein
Os02g0175850		Non-protein coding transcript
Os02g0175950		Non-protein coding transcript
Os02g0176300		Hypothetical protein
Os02g0176500		Hypothetical gene
Os02g0177633		Hypothetical protein
Os02g0177666		Hypothetical protein
Os02g0178250		Hypothetical protein
Os02g0178901		Non-protein coding transcript
Os02g0179225		Hypothetical protein
Os02g0179250		Hypothetical protein
	LOC_Os02g07850	DUF630/DUF632 domains containing protein, putative, expressed
	LOC_Os02g07940	Retrotransposon protein, putative, unclassified, expressed
	LOC_Os02g07950	Retrotransposon protein, putative, unclassified
	LOC_Os02g07990	Hypothetical protein
	LOC_Os02g08018	Calcium-transporting ATPase 9, plasma membrane-type, putative, expressed
	LOC_Os02g08020	Hypothetical protein
	LOC_Os02g08040	Expressed protein
	LOC_Os02g08050	Expressed protein
	LOC_Os02g08060	Expressed protein
	LOC_Os02g08210	Expressed protein
	LOC_Os02g08240	STE_PAK_Ste20_STLK.1-STE kinases include homologs to sterile 7, sterile 11 and sterile 20 from yeast, expressed

Table S9 The gene annotation within the maximum interval of *qSER-2b* identified from the databases of RAP and MSU

RAP-ID	MSU-ID	Gene annotation
Os02g0193200	LOC_Os02g09980	Expressed protein
Os02g0193300	LOC_Os02g09990	Expressed protein
Os02g0193400	LOC_Os02g10000	Zinc-binding protein, putative, expressed
Os02g0193500	LOC_Os02g10010	Expressed protein
Os02g0193600	LOC_Os02g10020	Mad3/BUB1 homology region 1 domain containing protein, expressed
Os02g0193732	LOC_Os02g10030	Expressed protein
Os02g0193800	LOC_Os02g10040	Guanine nucleotide-exchange protein like, putative, expressed
Os02g0193900	LOC_Os02g10050	Expressed protein
Os02g0194000	LOC_Os02g10060	SWIRM domain containing protein, expressed
Os02g0194100	LOC_Os02g10070	Citrate synthase, putative, expressed
Os02g0194200	LOC_Os02g10080	Zinc finger C-x8-C-x5-C-x3-H type family protein, expressed
Os02g0194300	LOC_Os02g10090	Expressed protein
Os02g0194400	LOC_Os02g10100	Leucine-rich repeat receptor protein kinase EXS precursor, putative, expressed
Os02g0194600	LOC_Os02g10110	Leucine Rich Repeat family protein, expressed
Os02g0194700	LOC_Os02g10120	Lipoxygenase, putative, expressed
Os02g0194800	LOC_Os02g10130	Tubulin binding cofactor C, putative, expressed
Os02g0195000	LOC_Os02g10160	AGAP003371-PA, putative, expressed
Os02g0195300	LOC_Os02g10180	DNA J homolog subfamily C member, putative, expressed
Os02g0195500	LOC_Os02g10190	DUF1680 domain containing protein, putative, expressed
Os02g0195600	LOC_Os02g10200	Zinc finger A20 and AN1 domain-containing stress-associated protein, putative, expressed
Os02g0195800	LOC_Os02g10220	Heat shock protein DnaJ, putative, expressed
Os02g0196000	LOC_Os02g10230	Metal cation transporter, putative, expressed
Os02g0196100	LOC_Os02g10240	ZOS2-05 - C2H2 zinc finger protein, expressed
Os02g0196300	LOC_Os02g10260	Outer membrane protein, OMP85 family protein, expressed
Os02g0196451	LOC_Os02g10280	Transposon protein, putative, unclassified, expressed
Os02g0196600	LOC_Os02g10290	Copper-transporting ATPase, putative, expressed
Os02g0196700	LOC_Os02g10300	Polygalacturonase, putative, expressed
Os02g0196800	LOC_Os02g10310	Fumarylacetoacetase, putative, expressed
Os02g0196900	LOC_Os02g10320	3-oxoacyl-synthase, putative, expressed
Os02g0197200	LOC_Os02g10350	MLO domain containing protein, putative, expressed
Os02g0197300	LOC_Os02g10360	MONOCULM 1, putative, expressed
Os02g0197400	LOC_Os02g10370	HrpN-interacting protein from Malus, putative, expressed
Os02g0197500	LOC_Os02g10380	Regulator of chromosome condensation domain containing protein, expressed
Os02g0197600	LOC_Os02g10390	Chlorophyll A-B binding protein, putative, expressed
Os02g0197700	LOC_Os02g10400	Expressed protein
Os02g0197750	LOC_Os02g10420	Expressed protein
Os02g0193350		Hypothetical conserved gene
Os02g0193425		Non-protein coding transcript
Os02g0193666		Non-protein coding transcript
Os02g0193933		Similar to predicted protein
Os02g0193967		Non-protein coding transcript
Os02g0194350		Hypothetical protein
Os02g0194900		BZIP transcription factor, putative, expressed
Os02g0195150		Hypothetical gene
Os02g0195533		Hypothetical protein
Os02g0195566		Non-protein coding transcript
Os02g0195900		Conserved hypothetical protein
Os02g0196750		Hypothetical protein
Os02g0196850		Hypothetical protein
Os02g0197050		Hypothetical gene
Os02g0197125		Hypothetical protein
	LOC_Os02g10140	BZIP transcription factor, putative, expressed
	LOC_Os02g10150	BZIP transcription factor, putative, expressed
	LOC_Os02g10170	Expressed protein
	LOC_Os02g10210	Expressed protein
	LOC_Os02g10250	Expressed protein
	LOC_Os02g10270	Retrotransposon protein, putative, unclassified, expressed
	LOC_Os02g10330	Retrotransposon protein, putative, unclassified, expressed
	LOC_Os02g10340	Retrotransposon protein, putative, unclassified, expressed
	LOC_Os02g10410	Expressed protein

Table S10 The gene annotation within the maximum interval of *qSER-3a* identified from the databases of RAP and MSU

RAP-ID	MSU-ID	Gene annotation
Os03g0352500	LOC_Os03g22900	SNF2 family N-terminal domain containing protein, expressed
Os03g0352566	LOC_Os03g22910	Expressed protein
Os03g0352600	LOC_Os03g22930	Expressed protein
Os03g0352700	LOC_Os03g22940	Expressed protein
Os03g0352800	LOC_Os03g22950	Acyl carrier protein, putative, expressed
Os03g0353000	LOC_Os03g22960	Expressed protein
Os03g0353300	LOC_Os03g22990	OsFBX84-F-box domain containing protein, expressed
Os03g0353333	LOC_Os03g23000	Expressed protein
Os03g0353400	LOC_Os03g23010	Expressed protein
Os03g0353500	LOC_Os03g23020	Expressed protein
Os03g0353600	LOC_Os03g23030	Aberrant pollen transmission 1, putative, expressed
Os03g0353900	LOC_Os03g23050	Expressed protein
Os03g0355100	LOC_Os03g23900	Expressed protein
Os03g0355200	LOC_Os03g23935	WD domain, G-beta repeat domain containing protein, expressed
Os03g0355300	LOC_Os03g23920	Expressed protein
Os03g0355500	LOC_Os03g23940	Expressed protein
Os03g0355600	LOC_Os03g23950	Adapitin protein, putative, expressed
Os03g0355700	LOC_Os03g23960	IQ calmodulin-binding motif family protein, putative, expressed
Os03g0355800	LOC_Os03g23970	Diphthine synthase, putative, expressed
Os03g0353366		Hypothetical gene
Os03g0354500		Domain of unknown function DUF834 domain containing protein
Os03g0355400		WD40 repeat-like domain containing protein
	LOC_Os03g22970	Retrotransposon protein, putative, Ty3-gypsy subclass
	LOC_Os03g22980	Retrotransposon protein, putative, Ty1-copia subclass
	LOC_Os03g23040	Expressed protein
	LOC_Os03g23060	Transposon protein, putative, unclassified, expressed
	LOC_Os03g23070	Retrotransposon protein, putative, unclassified, expressed
	LOC_Os03g23080	Retrotransposon protein, putative, unclassified, expressed
	LOC_Os03g23090	Retrotransposon protein, putative, Ty3-gypsy subclass
	LOC_Os03g23100	Retrotransposon protein, putative, Ty3-gypsy subclass
	LOC_Os03g23110	Retrotransposon protein, putative, Ty3-gypsy subclass, expressed
	LOC_Os03g23120	Retrotransposon protein, putative, Ty3-gypsy subclass, expressed
	LOC_Os03g23130	Retrotransposon protein, putative, unclassified
	LOC_Os03g23140	Retrotransposon protein, putative, unclassified
	LOC_Os03g23150	Retrotransposon protein, putative, Ty3-gypsy subclass, expressed
	LOC_Os03g23160	Retrotransposon protein, putative, Ty3-gypsy subclass, expressed
	LOC_Os03g23180	Retrotransposon protein, putative, Ty3-gypsy subclass, expressed
	LOC_Os03g23190	Retrotransposon protein, putative, unclassified
	LOC_Os03g23200	Retrotransposon protein, putative, Ty3-gypsy subclass, expressed
	LOC_Os03g23210	Retrotransposon protein, putative, unclassified, expressed
	LOC_Os03g23220	Retrotransposon protein, putative, Ty3-gypsy subclass, expressed
	LOC_Os03g23230	Retrotransposon protein, putative, unclassified
	LOC_Os03g23250	Retrotransposon protein, putative, unclassified, expressed
	LOC_Os03g23760	Retrotransposon protein, putative, Ty3-gypsy subclass, expressed
	LOC_Os03g23770	Retrotransposon protein, putative, unclassified, expressed
	LOC_Os03g23790	Retrotransposon protein, putative, Ty3-gypsy subclass, expressed
	LOC_Os03g23800	Retrotransposon protein, putative, Ty3-gypsy subclass, expressed
	LOC_Os03g23820	Retrotransposon protein, putative, unclassified
	LOC_Os03g23830	Retrotransposon protein, putative, Ty3-gypsy subclass, expressed
	LOC_Os03g23840	Retrotransposon protein, putative, Ty3-gypsy subclass, expressed
	LOC_Os03g23850	Retrotransposon protein, putative, Ty3-gypsy subclass
	LOC_Os03g23860	Retrotransposon protein, putative, Ty3-gypsy subclass, expressed
	LOC_Os03g23880	Retrotransposon protein, putative, unclassified
	LOC_Os03g23890	Retrotransposon protein, putative, Ty3-gypsy subclass

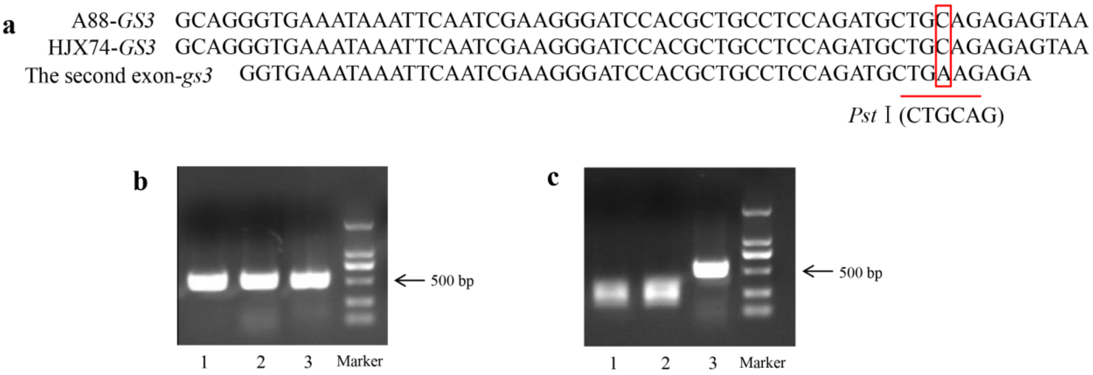

Fig. S1 Genotyping of the *GS3* gene in A88 and HJX74. **a**, The DNA sequence variation in functional site of *GS3*. **b**, Bands amplified from different samples using the SF28-*Pst* Ⅰ marker. **c**, Digested products of the amplified bands by *Pst* Ⅰ. Samples: 1, A88 (*GS3*); 2, HJX74 (*GS3*); 3, Control (*gs3*).

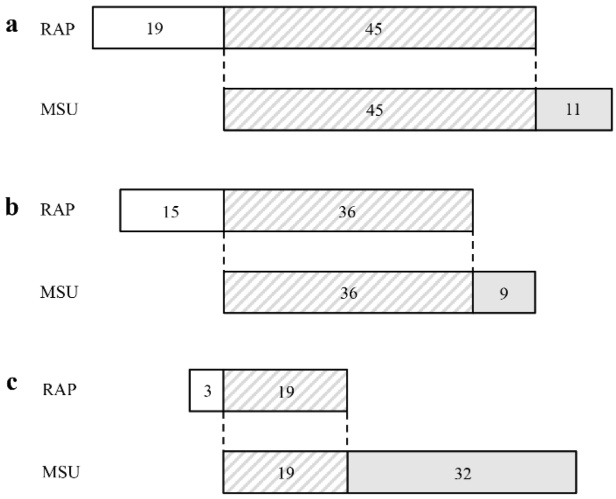

Fig. S2 Numbers of open reading frames (ORFs) identified within the maximum intervals of *qSER-2a* (**a**), *qSER-2b* (**b**) and *qSER-3a* (**c**). Shaded box is the ORF number identified from both the RAP-DB and the MSU-RAP. White box is the ORF number identified only from the RAP-DB. Gray box is the ORF number identified only from the MSU-RAP.
